# Diagnostic pathways and delay among tuberculosis patients in Stockholm, Sweden: a retrospective observational study

**DOI:** 10.1186/s12889-019-6462-5

**Published:** 2019-02-04

**Authors:** Anna Wikell, Helena Åberg, Jad Shedrawy, Isac Röhl, Jerker Jonsson, Ingela Berggren, Charlotte Buxbaum, Knut Lönnroth, Judith Bruchfeld

**Affiliations:** 1 Division of Infectious Diseases, Department of Medicine, Karolinska Institutet, 171 77 Stockholm, Sweden; 2 Department of Public Health Sciences, Karolinska Institutet, 171 77 Stockholm, Sweden; 3 The Public Health Agency of Sweden, 171 65 Solna, Sweden; 4 Department of Communicable Disease Control & Prevention, Stockholm County Council, 118 91 Stockholm, Sweden; 5 Department of Pediatrics, Karolinska University Hospital Huddinge, 141 86 Stockholm, Sweden; 6 Centre for Epidemiology and Community Medicine, Stockholm County, 104 31 Stockholm, Sweden; 7 Department of Infectious Diseases, Karolinska University Hospital Solna, 171 76 Stockholm, Sweden

**Keywords:** Asylum seekers, Diagnostic delay, Health examination, Screening, Tuberculosis

## Abstract

**Background:**

Asylum seekers in Sweden are offered tuberculosis (TB) screening at a voluntary post-arrival health examination. The role of this screening in improving the TB diagnostic pathway has not been previously evaluated. The aim of this study was to determine diagnostic pathways for active TB cases and compare diagnostic delays between different pathways.

**Methods:**

Retrospective review of medical records of patients reported with active TB in Stockholm in 2015, using a structured and pre-coded form.

**Results:**

Seventy-one percent of patients actively sought health care due to symptoms. As for source of referral to TB specialist clinic, 15% came from screening of eligible migrants, of whom the majority were asymptomatic. Among asylum seekers, 69% were identified through screening at a health examination (HE). The main sources of referral to TB clinics were emergency departments (27%) and primary health care centers (20%). Median health care provider delay was significantly longer in patients identified through migrant screening in health examination.

**Conclusions:**

Screening at a health examination was the main pathway of active TB detection among mainly asymptomatic and non-contagious asylum seekers but contributed modestly to total overall TB case detection. In these patients TB was diagnosed early in a non-contagious phase of the disease. Further research is required to assess the effectiveness and cost-effectiveness of HE TB screening as well as inclusion of other groups of migrants from high incidence countries in the screening program in terms of impact on delay, transmission and treatment outcomes.

**Electronic supplementary material:**

The online version of this article (10.1186/s12889-019-6462-5) contains supplementary material, which is available to authorized users.

## Background

In recent years tuberculosis (TB) epidemiology in many low-incidence countries (countries with a TB incidence of < 10/100,000), including Sweden, has changed due to increased globalization and migration [1–3]. The proportion of notified TB cases that were foreign born increased from 50 to 60% between 2009 and 2015 in those countries. [3]

Early diagnosis and treatment of active TB is essential to improve health outcomes and reduce transmission. Systematic screening for active TB can potentially increase case notification and reduce diagnostic delays although the evidence is conflicting. Few studies have specifically assessed if systematic TB screening can shorten the pathway to diagnosis, compared to the conventional diagnostic pathways starting with symptoms that leads to patients actively seeking care (so-called “passive case finding”) [4, 5]. Acknowledging the weak evidence base, the World Health Organization (WHO) conditionally recommends that migrants from high-incidence to low-incidence countries should be considered as target groups for systematic screening for both active TB and latent TB infection (LTBI) [6–8]. These guidelines are part of the WHO ‘End TB strategy’ and its adaptation to low-incidence countries, which includes targets of a 90% TB incidence reduction and a 95% reduction in TB deaths by 2035 [1, 9]. The weak evidence might explain the very heterogeneous migrant TB screening policies across European countries with similar TB epidemiology [10].

In Sweden, the TB notification rate in the foreign-born population increased by 18% between 2009 and 2015, while it decreased by 24% among the Swedish-born. As a consequence, the proportion of notified TB cases that were foreign born increased from 73 to 90% during this period [3]. In 2015, an estimated 11% of TB cases were likely to have been infected in Sweden [11]. This figure increased to 16% in 2016 [12, 13], possibly due to the massive increase in asylum seekers in 2015 and resulting delays in the TB screening process. [14]

In Sweden, all asylum seekers, as well as quota refugees and some reunified family members including all children of school age are invited to a voluntary post-arrival health examination (HE), within which TB screening is also performed, predominantly for individuals from countries with a TB incidence of over 100 cases per 100,000 [15, 16]. Asylum seekers who have received refugee status are eligible for a HE up to 2 years after arrival. Migrants coming for other reasons such as studies or work as well as most late-arriving relatives are not invited to a HE.

Little has previously been done to evaluate the effectiveness of the Swedish TB screening programme. It is not known if TB screening at HE in Sweden reduces diagnostic delay of active TB, contributes significantly to case detection or effectively reduces TB burden. All active TB cases in Sweden are reported to county medical authorities and the national public health agency. However, diagnostic pathways or diagnostic delays are not well documented in the routine notification system. Therefore, this study aimed to: i) determine first health care contact and diagnostic pathway distribution for active TB cases, ii) determine the contribution of TB screening in HE to overall case detection, iii) compare diagnostic delays by source of referral to TB specialist clinic, and iv) compare health care provider delay in patients detected through TB screening at HE and patients detected through non-screening pathways.

## Methods

### Study design and study population

This was a retrospective observational study. Study subjects included all patients who were diagnosed with active TB within Stockholm County and reported to the Department of Communicable Disease Control and Prevention, Stockholm, during 2015. A list of diagnosed patients’ personal identifiers was used to access medical records in the electronic medical system Take Care at the TB Centers, Departments of Infectious Diseases and Paediatrics at Karolinska University Hospital, which manage all TB cases in Stockholm County. The same electronic medical record system is used in primary care and data are linkable.

### Study setting and screening policy

**Fig. 1:**
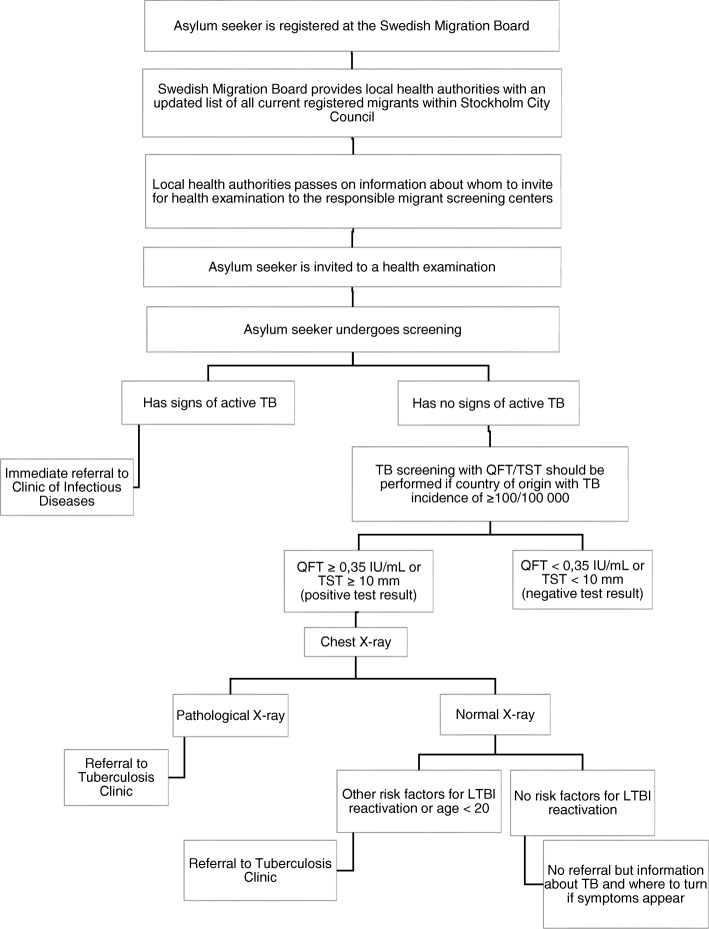
Cascade of care in TB screening among asylum seekers according to Stockholm County Council guidelines (17–19) *Abbreviations*: *TB – tuberculosis, QFT – QuantiFeron, TST – tuberculin skin test, LTBI – latent tuberculosis infection, ED - Emergency Department*

The responsibility for performing HE for eligible migrants in Stockholm has been allocated to seven specific primary health care centres, here referred to as screening centres [16]. TB symptom screening should be done for all, while QuantiFERON TB Gold (QFT) (more recently Quantiferon Plus) should be performed for persons with recent TB contact and persons from countries with high TB incidence (≥100 cases per 100,000). [17, 18] If TB symptoms are present at screening the patient should be referred immediately and preferably seen the same day at an emergency department (ED). A positive QFT test in an asymptomatic person should be followed by a chest X-ray (CXR). If (CXR) is pathological or if a risk factor for progression to active TB is present, the patient should be referred for specialist assessment. Risk factors for development of active TB that should lead to referral for possible LTBI treatment are age below 20, disease associated with immunosuppression, current pregnancy or delivery within the last 6 months. [19] Asymptomatic patients referred to a specialist physician are usually first seen by a TB nurse for further symptom screening and if active TB suspicion also asked to provide sputum samples for mycobacterial analysis. When results are available further diagnostic work-up is decided by the treating physician. For detailed recommended cascade of care, see Fig. 1 [18–20]. Through this pathway, active TB should be detected early, preferably before further transmission has occurred. In addition, detection and treatment of LTBI should prevent reactivation among high-risk groups. Most LTBI reactivations occur within 2 years after infection [20].

### Data collection and analysis

Data was extracted from electronic patient records using a structured and pre-coded form. A verified diagnosis was defined as *Mycobacterium tuberculosis* (*Mtb*) findings in microscopy, PCR or culture. A diagnosis without microbiological verification was usually based on symptoms, radiographic imaging or histopathological analysis and categorized as a clinical diagnosis.

Country of origin was categorized into high TB incidence countries (≥100/100000) or others (< 100/100000). To assess non-screening diagnostic pathways, data on first health care contact after onset of symptoms, and source of referral to TB specialist clinic, were collected. ‘Symptoms’ include any symptoms later deemed to be caused by TB according to the medical record.

**Fig. 2:**
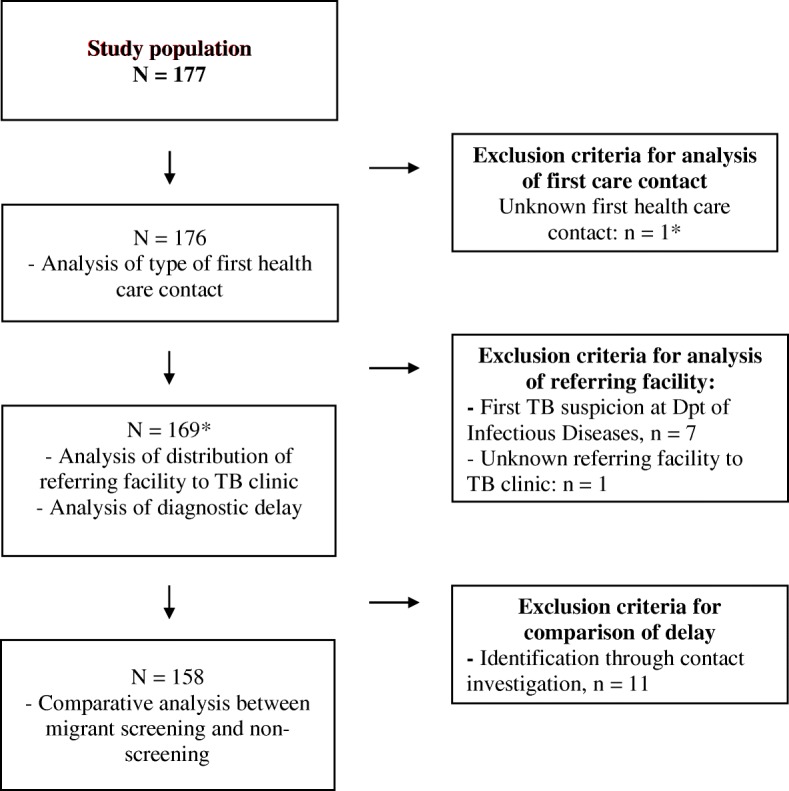
Flowchart of exclusion criteria per analysis* The patient excluded due to missing data on first health care facility contact was re-included for the subsequent analysis. Therefore the number of patients included in analysis of distribution of referring facility to TB clinic and analysis of diagnostic delay is calculated as 176 + 1–7 – 1 = 169

Inclusion and exclusion of patients in the study sample and sub-analyses are shown in Fig. 2. 177 patients were diagnosed with TB within Stockholm county and reported to the Department of Communicable Disease Prevention and Control during 2015. For analysis of first health care contact, one patient was excluded due to unknown first health care contact. For analysis of distribution of sources of referral and delay per source of referral, patients for whom first TB suspicion was at Department of Infectious Diseases were excluded (since this was the referral destination in this analysis), leaving 169 participants included in this analysis. For the analysis comparing patient characteristics and delay in the HE TB screening pathway group to the non-screening pathway group, further exclusion was made of the 11 patients identified through contact investigation since they represent a different type of screening pathway (i.e. TB screening in close contacts to active TB cases). Consequently, 158 patients were included in these analyses.

For non-screening groups the total diagnostic delay was defined as number of days from onset of TB symptoms to TB treatment initiation, which is the sum of the number of days from onset of symptoms to first health care contact (patient delay) and the number of days from first health care contact to TB treatment initiation (health care provider delay). Delay endpoint was set to date of treatment initiation due to difficulties finding adequate dates of diagnosis for clinically diagnosed patients. For asymptomatic persons screened for TB patient delay was set to zero days. Comparison of health care provider delay was performed between the screening and non-screening groups. The proportion with bacteriologically confirmed TB was compared between the groups.

For data analysis and statistical calculations, Excel and IBM® SPSS Statistics® version 24 were used. Regarding diagnostic delays, descriptive statistical analysis of medians with interquartile range (IQR) were calculated. Median values were used for diagnostic delay due to skewed data. Statistical significances of differences regarding categorical variables were calculated using Chi-Square test. Continuous data were tested for normal distribution using Shapiro-Wilk test. For non-normally distributed data, non-parametric Mann-Whitney U test was performed. Statistical significance regarding differences in missing data was tested using independent T-test (age), Pearson’s chi-square (gender) and Fisher’s exact test (country of origin). A confidence interval of 95% was used and *p* < 0.05 was deemed significant. Adjustment for variables gender and age regarding health care provider delay was initially performed using linear regression, followed by multivariable logistic regression with health care provider delay dichotomized at median as the outcome. B coefficients, *P* values, odds ratios and 95% confidence intervals for odds ratio were calculated. We chose not to control for country of origin due to insufficient subgroup size per individual country.

## Results

Of the 177 patients included in at least one of our analyses, 111 (63%) were men. Forty-one patients (23%) were under 20 at time of diagnosis, 9 (5%) were aged under 15. One-hundred and fifty-two patients (86%) were foreign-born and 89 (50%) were from a country with TB incidence ≥100/100. 000. Thirty-six (20%) were noted as asylum seekers in the medical record at time of TB investigation (Table 1). Patient characteristics of the health examination screening subgroup and the non-screening subgroup respectively are presented in Table 2.

**Table 1:** Study population characteristics

Variable No. (%)	Study population *n* = 177
Gender	
Male	111 (63)
Female	66 (37)
Age at diagnosis	
0–10	1 (0)
11–20	42 (24)
21–30	42 (24)
31–40	40 (22)
41–50	26 (15)
51–100	26 (15)
TB incidence in country of origin	
≥ 100/100000	89 (50)
< 100/100000	88 (50)
Asylum seekers at time of investigation^§^	36 (20)
Previous TB diagnosis, classification	
Active TB	14 (8)
Latent TB	6 (3)
Country of birth*	
Eritrea	27 (15)
Sweden	25 (14)
Somalia	19 (11)
Afghanistan	18 (10)
Ethiopia	7 (4)
Other (36 different)	81 (46)

*Abbreviations: TB = tuberculosis,* ^§^ 85 participants (48%) had unknown status. 2 of the 25 participants referred from migrant TB screening at HE had unknown status. 1 was a reunified family immigrant

*The countries listed are the most frequent countries of birth among all patients

**Table 2:** Characteristics of health examination screening subgroup and non-screening subgroup, *n* = 158

Variable No. (%)	*Sub-group 1: HE TB screening group n* = 25	Sub-group 2: Non-screening group** n = 133
Gender		
Male	20 (80)	84 (63)
Female	5 (20)	49 (37)
Age at diagnosis		
0–10	0 (0)	1 (1)
11–20	19 (76)	18 (14)
21–30	3 (12)	33 (25)
31–40	3 (12)	33 (25)
41–50	0 (0)	24 (18)
51–100	0 (0)	24 (18)
TB incidence in country of origin		
≥ 100/100000	16 (64)	56 (42)
< 100/100000	9 (36)	77 (58)
Asylum seekers at time of investigation^§^	22 (88)	12 (9)
Previous TB diagnosis, classification		
Active TB	1 (4)	10 (8)
Latent TB	0 (0)	5 (4)
Country of birth*		
Eritrea	9 (36)	17 (13)
Sweden	0 (0)	24 (18)
Somalia	3 (12)	11 (8)
Afghanistan	8 (32)	7 (5)
Ethiopia	2 (8)	4 (3)
Other (36 different)	3 (12)	70 (53)

*Abbreviations: HE =* health examination*, TB =* tuberculosis*,*^§^ 85 participants (48%) had unknown status. 2 of the 25 participants referred from migrant TB screening at HE had unknown status. 1 was a reunified family immigrant

*The countries listed are the most frequent countries of birth among all patients

**Definition of “non-screening group”: Patients identified through primary health care visits, emergency department, or other in- or outpatient clinics other than clinic of infectious diseases. Excluded from this group are 11 patients identified through contact investigation (a separate type of screening), 7 patients identified at Clinic of infectious diseases and 1 patient with missing data of referring clinic

**Fig. 3:**
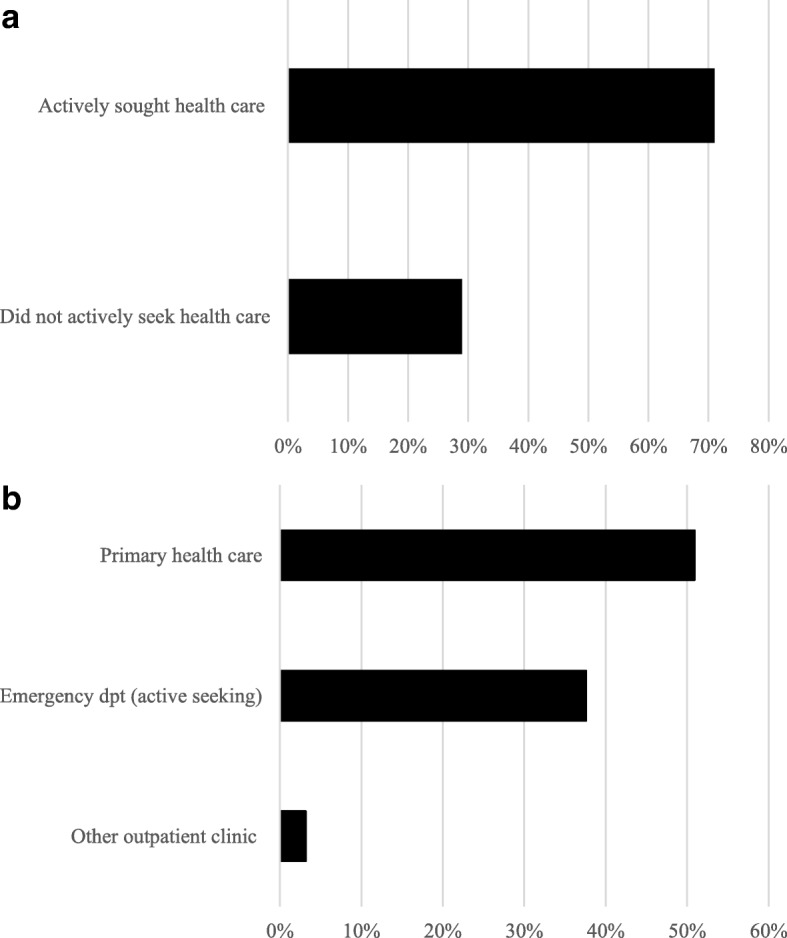
**a** Distribution of active care seeking vs. non-active (screening pathway, including contact investigation)), *n* = 176. **b** Distribution of first health care contacts among patients who actively sought health care, *n* = 125 Excluded patients: - *Patients with missing data on first health care contact, n = 1* - *Only in 3b: Patients who did not actively seek health care for tuberculosis symptoms, n = 51*

One-hundred and twenty-five (71%) out of 176 patients had actively sought health care due to symptoms (Fig. 3a). Distribution of first health care contacts in the non-screening group is shown in Fig. 3b.

**Fig. 4:**
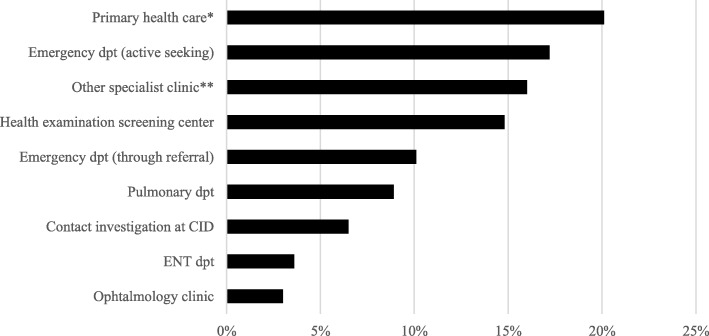
Sources of referral to tuberculosis clinic among active tuberculosis patients diagnosed 2015 in Stockholm, percentages. *Abbreviations: Dpt = department, CID = clinic of infectious diseases, ENT = ear, nose, throat. N = 169.* *Primary health care visit not including migrant screening in health examination. **‘Other specialist clinic’ include for example urology ward, internal medicine ward, rheumatology ward, maternity clinic and pediatric ward Excluded patients: - *Patients identified through visit at clinic of infectious diseases (not contact investigation), n = 7* - *Missing data on source of referral to TB specialist clinic, n = 1*

As for source of referral to TB specialist clinic, 25 (15%) out of 169 patients were identified through migrant TB screening at HE and 11 (7%) were identified through contact investigation. Emergency department (27%, 46 out of 169 patients) and primary health care centers (PHC) (20%, 34 patients) were the most common health care providers referring patients to TB centers (Fig. 4). Among all 40 patients aged below 20 years of age at time of investigation, 19 (48%) were referred to TB clinic from TB screening at HE.

Thirty-six study participants were recorded as asylum seekers by the time of investigation. Twenty-four of these were first suspected having TB in screening at HE, five in PHC, three in the ED, three in other specialist clinic and one through contact investigation.

In the HE TB screening group of asylum seekers, 20 (91% of the 22 referred from HE after TB screening) had no symptoms at screening. These were referred due to positive QFT or tuberculine skin test (TST) combined with pathological CXR and/or risk factor. Two patients (8%) were immediately referred due to suspected symptoms of active TB.

Thirty-three asylum seeker patients (92% of all 36 asylum seeker participants) had been invited for HE and 31 (86% of all 36) attended. Thirty-one (86%) of those were from high TB incidence countries and should consequently undergo TB screening. Two patients were already on TB treatment by time of screening. Thus, 29 were eligible for TB screening and all of them were screened. Twenty-four of these also underwent CXR due to positive screening tests. Two of the 29 tested had positive test result, but were not referred for CXR. Two participants had a negative test result and one had no noted test result in the medical chart. Among 24 patients fulfilling criteria for referral to TB specialist clinic, 23 (96%) were referred. One patient was referred although not fulfilling criteria by the time. One referral was never responded to, why the patient eventually was referred from the ED instead. For one patient who fulfilled referral criteria, the referral from HE was not sent until several weeks after receiving test results. In the meantime, the patient was referred to TB specialist clinic from PHC.

Median health care provider delay was 54 days among HE screening patients and 26 days among non-screening patients and this difference was statistically significant (*p* < 0.001) (Table 3). There was no significant association between age or gender, respectively, and long health care delay (for further details on multivariable analysis, see in Additional file 1: Table S1). There were no significant differences in age, sex, or country of origin between those with and without information about delay. The proportion of cases that was bacteriologically confirmed was 56% in the HE screening group, and 86% in the non-screening group (Table 3). Patient delay was by definition set to zero for the non-symptomatic patients that were screened at HE (92% of them). Among non-screened patients, median patient delay was 21 days.

**Table 3:** Comparison of clinical characteristics and health care provider delay between the health examination screening subgroup and non-screening subgroup, *n* = 158

Variable	*Subgroup 1:* *HE TB screening* (*n* = 25)	*Subgroup 2: Non-screening group* (*n* = 133)	Statistical significance (2-tailed)
Age at diagnosis			
Median (IQR)	16 (15–21)	36 (26–47)	p < 0.001*
Type of TB, No. (%)			
Pulmonary (*n* = 93)	17 (68)	76 (57)	*p* = 0.379†
Extrapulmonary (*n* = 44)	5 (20)	39 (29)	*p* = 0.340†
Extrapulmonary with lung engagement (*n* = 12)	2 (8)	10 (8)	*p* = 0.934†
Disseminated (n = 3)	1 (4)	2 (1)	*p* = 0.401†
Miliary (*n* = 6)	0 (0)	6 (5)	*p* = 0.279†
Type of diagnostic method, No. (%)			
Microbiologically verified‡ (*n* = 128)	14 (56)	114 (86)	*p* = 0.001†
Clinical§ (*n* = 30)	11 (44)	19 (15)	
Microbiologically verified TB with pulmonary engagement, No. (%) (*n* = 100)	12 (48)	88 (66)	p < 0.001†
Median health care provider delay in days (IQR)	54 (38–106), *n* = 22	26 (7–66), *n* = 121	*p* < 0.001*

*Mann-Whitney U test, † Chi-Square test

*Abbreviations: HE =* health examination*, TB =* tuberculosis

*Definitions:*

‡ = *Mycobacterium tuberculosis* findings in microscopy, PCR or culture

§ = Diagnosis without microbiological verification, based on symptoms, imaging or histopathological analysis

*Excluded patients:*

- Patients identified through contact investigation, *n* = 11

- Patients identified through visit at clinic of infectious diseases, *n* = 7

- Patient with missing source of referral data, *n* = 1

Within the non-screening group, the median total delay was 30 days among patients referred to TB centers from ED, which was significantly shorter compared to the 85 days for those referred from PHCs (non-HE screening group) (p < 0.001). Median patient delay for the ED group was 8 days as compared to 31 days in the PHC group (*p* = 0.002). Median health care delay for ED group was 9 days as compared to 24 days among the PHC patients (*p* = 0.022) (Table 4).

**Table 4:** Diagnostic delays by source of referral among patients diagnosed with active tuberculosis in Stockholm, 2015, *n* = 169

Source of referral to TB specialist clinic	Patient delay (days): median (IQR)	Health care provider delay (days): median (IQR)	Total delay (days): median (IQR)
Primary health care centre (*n* = 34)	31 (19–98) (*n* = 32)*	24 (5–62) (*n* = 33)	85 (42–149) (*n* = 31)
Other specialist facilities (*n* = 53)	13 (3–31)(*n* = 41)	57 (19–113) (*n* = 43)	85 (38–132) (*n* = 38)
Emergency dpt (actively sought) (*n* = 29)	8 (3–45) (*n* = 27)	9 (3–24) (*n* = 28)	30 (14–53) (*n* = 27)
Emergency dpt (through referral) (*n* = 17)	28 (6–37) (*n* = 15)	29 (11–51) (*n* = 17)	65 (34–91) (*n* = 15)
Health examination screening center (*n* = 25)	–	54 (38–106) (*n* = 22)	–
Contact investigation at CID (*n* = 11)	–	48 (24–62) (*n* = 11)	–

*Abbreviations: TB* = tuberculosis, *SD* = standard deviation, *IQR* = interquartile range,

*dpt* = department, *CID* = clinic of infectious diseases

*The numbers noted in each box are the number of patient with available delay data per subgroup

*Excluded patients:*

- Patients identified through visit at clinic of infectious diseases, *n* = 7

- Patient with missing source of referral data, *n* = 1

## Discussion

The majority of patients eligible for TB screening at HE had been investigated and referred according to guidelines and the majority of asylum seekers with TB had indeed been detected through HE TB screening. The screening policy thus seems well implemented for those who are eligible.

However, TB screening at HE contributed only 15% of all detected cases. Fifty percent of all cases were born in a high TB incidence country, while 20% were asylum seekers at the time of investigation. TB screening of migrants in its current design in Sweden is by policy limited mainly to asylum seekers, leading to missed opportunities for early case detection and prevention among other migrants from high-incidence countries, such as guest students or labor migrants. Based on our data, it could be argued that other migrants from high-incidence countries should be included in the migrant screening program upon arrival to Sweden, particularly considering that most TB reactivations in this group occur within 5 years after arrival [21]. To partly address this problem, all pregnant women from high TB incidence countries are since 2016 screened for TB at maternity clinics in Stockholm County. [20]

Patients detected through HE TB screening were mainly asymptomatic but were referred based on a positive QFT screening test with or without pathological CXR, and were thus often initially handled as non-prioritized possible latent TB cases prior to assessment at the specialized TB center. This may explain the finding that the health care provider delay was significantly longer in the HE screening group compared to the non-screening group. The higher proportion of non-microbiologically confirmed cases in the HE screening group may indicate early diagnosis of less advanced and non-contagious TB through screening. However, it is also possible that some clinically diagnosed cases did not have active TB. From our data it is not possible to conclude if TB screening at HE shortens total diagnostic delays.

Passive case finding with symptoms seeking at PHCs or EDs were the main diagnostic pathways. Diagnostic delay was significantly longer through PHCs than through EDs. The high median total delays could be due to the tendency of long health care delay for patients with short patient delay (no or mild symptoms) and vice versa. To shorten delay health staff in PHCs need to apply a higher index of TB suspicion in risk groups. ED staff in settings with Infectious Diseases specialists at the ED seem sensitized to suspect TB early.

Our study has some limitations. All patients diagnosed with active TB in Stockholm 2015 were included. This is a small group, although they represent 23% of all cases in Sweden. Another limitation was the retrospective design with a risk of selection bias by including only those with a recorded diagnosis of active TB. Data from medical records may be unreliable, especially exact timing of symptom onset. However, through the linkable medical record system we could establish the timing of all health care encounters in detail. Sample size of subgroups (asylum seeker group and age below 20 group) were small which limited statistical power.

## Conclusions

TB screening at a health examination was the main pathway of active TB detection among asylum seekers and performed well but contributed modestly to total overall TB case detection. In these mainly asymptomatic patients TB was diagnosed early in a non-contagious phase of the disease. Inclusion of other groups of migrants from high incidence countries in the screening program would increase the contribution of screening to case detection. The long health care provider delay in primary health care indicates a need for a higher index of suspicion of active TB in migrants from TB endemic countries. Further research is required to assess the effectiveness and cost-effectiveness of HE TB screening as well as inclusion of other groups of migrants from high incidence countries in the screening program in terms of impact on delay, transmission and treatment outcomes. [3–7, 22]

## Additional file

Additional file 1: **Table S1.** Data on multivariable analysis of health care provider delay Table data on multivariable analysis show that there was no significant association between age or gender, respectively, and long health care delay. (DOCX 47 kb)

## Abbreviations

CID: clinic of infectious diseases
CXR: chest X-ray
ED: emergency department
HE: health examination
LTBI: latent tuberculosis infection
PHC: primary health care center
QFT: QuantiFERON TB Gold
TB: tuberculosis
TST: tuberculin skin test
