# Supplementary material for: Diagnostic pathways and delay among tuberculosis patients in Stockholm, Sweden: a retrospective observational study
Source: BMC Public Health. 2019 Feb 4;19:151. doi: 10.1186/s12889-019-6462-5 (PMC6360687; doi:10.1186/s12889-019-6462-5)
Supplement: Supplementary file 1 — Table S1. Data on multivariable analysis of health care provider delay Table data on multivariable analysis show that there was no significant association between age or gender, respectively, and long health care delay. (DOCX 47 kb) [file 12889_2019_6462_MOESM1_ESM.docx]

**Supplementary material**

**Table 1: Data on multivariable analysis of health care provider delay**

| ***Dependent variable*** | | | | |
| --- | --- | --- | --- | --- |
| ≥ Median health care provider delay (33 days) | | | | |
| ***Independent variables*** | ***B coefficient*** | ***P value*** | ***Odds Ratio*** | ***95% CI for OR*** |
| **Gender** | 0.358 | 0.313 | 1.430 | 0.714 - 2.865 |
| **Age** | -0.002 | 0.809 | 0.998 | 0.978 - 1.017 |

*Abbreviations: CI = confidence interval, OR = odds ratio*
